# Influence of spatial configurations on electromagnetic interference shielding of ordered mesoporous carbon/ordered mesoporous silica/silica composites

**DOI:** 10.1038/srep03252

**Published:** 2013-11-19

**Authors:** Jiacheng Wang, Hu Zhou, Jiandong Zhuang, Qian Liu

**Affiliations:** 1 State Key Laboratory of High Performance Ceramics and Superfine Microstructure, Shanghai Institute of Ceramics, Chinese Academy of Science, Shanghai 200050, P. R. China; 2 University of Chinese Academy of Sciences, Beijing 100049, P. R. China

## Abstract

Ordered mesoporous carbons (OMCs), obtained by nanocasting using ordered mesoporous silicas (OMSs) as hard templates, exhibit unique arrangements of ordered regular nanopore/nanowire mesostructures. Here, we used nanocasting combined with hot-pressing to prepare 10 wt% OMC/OMS/SiO_2_ ternary composites possessing various carbon mesostructure configurations of different dimensionalities (1D isolated CS41 carbon nanowires, 2D hexagonal CMK-3 carbon, and 3D cubic CMK-1 carbon). The electric/dielectric properties and electromagnetic interference (EMI) shielding efficiency (SE) of the composites were influenced by spatial configurations of carbon networks. The complex permittivity and the EMI SE of the composites in the *X*-band frequency range decreased for the carbon mesostructures in the following order: CMK-3-filled > CMK-1-filled > CS41-filled. Our study provides technical directions for designing and preparing high-performance EMI shielding materials. Our OMC-based silica composites can be used for EMI shielding, especially in high-temperature or corrosive environments, owing to the high stability of the OMC/OMS fillers and the SiO_2_ matrix. Related shielding mechanisms are also discussed.

Recently, electromagnetic (EM) pollution from the widespread use of electronic devices has attracted great attention, prompting the exploration of effective EM shielding materials^1^,^2^. EM shielding materials are used to protect sensitive workspaces, environments, and circuits from EM radiation from telecommunication devices^3^. Various carbon-based materials with different structures, as well as their composites, including carbon black (CB)^4^,^5^, carbon nanofibers (CNFs)^6^,^7^,^8^, carbon nanotubes (CNTs)^4^,^9^,^10^,^11^,^12^, and graphene^13^,^14^,^15^, have attracted attention as EM shielding or absorbing materials because of their variable morphology, light weight, adjustable porosity, high conductivity, resistance to corrosion, and ease of processing.

The EM interference (EMI) shielding effectiveness (SE) of a composite is strongly related to several important factors, such as the intrinsic texture (*e.g.*, the shape, special distribution, and aspect ratio) of the conductive filler, thickness, electric conductivity, dielectric constant, and environmental temperature^16^,^17^. Cao and Yuan studied the effect of temperature on EMI shielding and microwave absorption performance of CNT/silica composites and short carbon fiber/silica composites over a frequency range of 8.2–12.4 GHz^18^,^19^. Notably, nanocarbon-filled SiO_2_ composites can be used as EMI shielding materials in harsh working environments, including high-temperature and corrosion conditions, offering advantages over many recently reported polymer-based shielding materials. Many studies have also investigated the influence of CNT aspect ratio on the EMI SE of CNT-filled composites^4^,^10^,^16^,^17^,^20^. For example, Li and coworkers demonstrated that single-wall CNT-polymer composites with high CNT aspect ratios significantly enhance EMI SE^10^. The high aspect ratio of the conductive filler provides a large interfacial area, favoring electron transport and electric conductivity. According to EM transmission line theory, this improves the EMI SE of composites. However, the most serious obstacle for the application of these composites is that CNT with high aspect ratios tend to tangle together and are thus difficult to disperse homogeneously throughout the matrix^21^. To overcome this problem, we adopted a method for in-situ nanocasting of ordered mesoporous silica (OMS)^22^,^23^, SBA-15, with carbon to obtain highly ordered carbon nanowire arrays to fill the mesopores of CMK-3^24^. This CMK-3/SBA-15 intermedium has an inter-filled mesostructure and a rod-like morphology with a limited length. Therefore, the conductive CMK-3/SBA-15 particles used to absorb the electromagnetic waves are easily dispersed in the SiO_2_ inorganic matrix. The resulting composite, CMK-3/SBA-15/SiO_2_, contains highly ordered carbon nanowire arrays and exhibits much better EMI SE in the *X*-band frequency range than CNT composites with SiO_2_ containing the same carbon content. This is due to better dispersion of the electromagnetic wave–absorbing materials in the matrix^25^. This finding motivates the present study on how the mesostructural spatial configurations of OMCs affect the EMI shielding performance of OMC-filled composites. OMCs can be produced with various spatial configurations and different dimensionalities (1D, 2D, and 3D) using different hard templates for replication, allowing the production of highly conductive networks that extend in different directions for electromagnetic energy exhaustion through movement of electrons. It has been reported that OMCs with different mesostructure configurations exhibit distinct properties that can be used in many applications, including adsorption of large molecules, chromatography, electrodes, and electrochemical energy storage devices^26^. In the field of EMI shielding, only OMCs with 2D hexagonal mesostructures, such as Fe-containing OMCs and OMC-alumina composites^27^,^28^, have been investigated. More recently, we reported that 2D CMK-3-filled poly(methyl methacrylate) (PMMA) composite films and in-situ surface-modified CMK-3/PMMA composites have great potential for use in EMI shielding and microwave absorption in the *X*-band frequency range^29^,^30^. However, the effects of OMC mesostructure configuration in different dimensions (1D, 2D, and 3D) on the EMI SE of OMC-loaded composites have rarely been reported. It is therefore important to study the relationship between different OMC mesostructure configurations and the EMI SE of OMC-loaded composites to provide guidance for the design and preparation of novel carbon-filled composites for EMI shielding.

In this work, we report the synthesis of inter-filled OMC/OMS structures with different mesostructure configurations embedded in a SiO_2_ matrix. We investigate the influence of the carbon mesostructures on the electric conductivity, complex permittivity, and EMI shielding properties of the resulting bulk composites. Different OMC mesostructures can be produced by changing the OMS template. As shown in Figure 1, three types of OMSs (3D cubic MCM-48, 2D (quasi-3D) hexagonal SBA-15, and 2D hexagonal MCM-41) were used as hard templates and filled with carbon to form OMC/OMS intermedia with inter-filled mesostructures^26^. These OMC/OMS intermedia were added into SiO_2_ sources to form dense ternary composites of OMC/OMS/fused silica with a calculated loading of 10 wt% carbon using a rapid sol-gel method in combination with hot-press sintering. The electric conductivity and dielectric and microwave attenuation properties at a frequency range of 8.2–12.4 GHz (generally known as the *X*-band, which is widely used in military and commercial fields) of these dense OMC/OMS/fused silica composites were then tested. We also examined the influence of OMC mesostructures on the properties of the composites and investigated the possible mechanisms.

## Results

**Figure 2:**
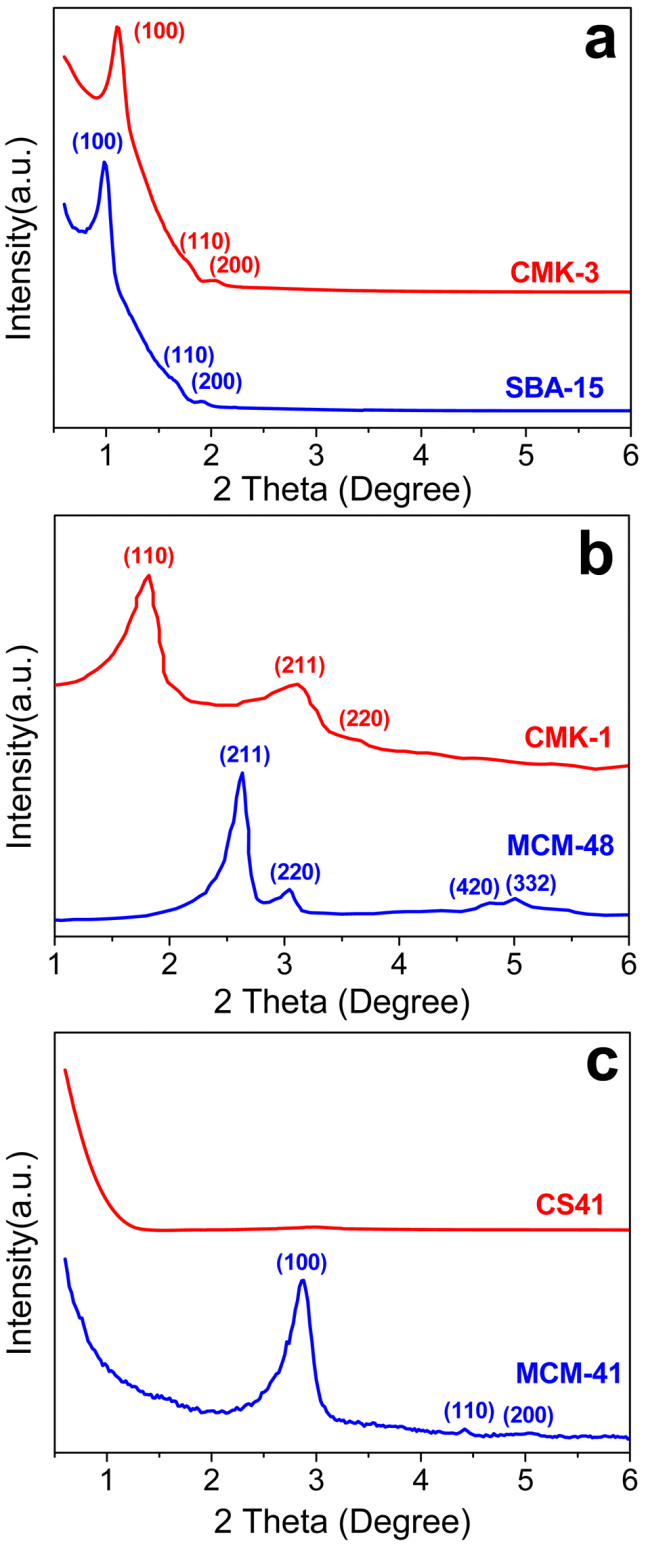
Small-angle XRD patterns for various OMS templates and the corresponding naked carbon replicas after the OMS templates were removed. (a) SBA-15 and CMK-3, (b) MCM-48 and CMK-1, and (c) MCM-41 and CS41.

To compare the textures of the dispersed OMCs within the matrix, the OMC/OMS intermedia, prepared by in-situ filling various OMSs (SBA-15, MCM-48, and MCM-41) with carbon, were stirred in an aqueous solution of 5 wt% hydrogen fluoride to etch off the OMS templates, obtaining the corresponding naked OMC (CMK-3, CMK-1, and CS41) replicas^26^. Figure 2 shows the small-angle X-ray diffraction (XRD) patterns of the OMS templates and their corresponding naked OMC replicas. SBA-15 and MCM-41 exhibit ordered 2D hexagonal structures (space group, *p*6*mm*)^31^,^32^, but MCM-48 exhibits a well-ordered 3D cubic structure (space group, *Ia*3*d*)^33^. The pure CMK-3 replica from SBA-15 has three well-resolved peaks ascribed to the (100), (110), and (200) reflections in the XRD pattern^34^, indicating a 2D hexagonal ordered arrangement (space group, *p*6*mm*) (Figure 2a). The diffraction peaks in the pattern for CMK-3 shift to a higher angle compared to its parent template SBA-15 owing to contraction of both the carbon framework and the silica template during the high-temperature carbonization process (900°C). The XRD pattern for CMK-1 differs from its template MCM-48. The presence of three well-defined diffractions, (110), (211), and (220), indicates a 3D cubic space lattice (space group, *I*4/*a*) for CMK-1 (Figure 2b). There is no diffraction in the XRD pattern for the CS41 carbon, indicating a non-ordered mesostructure for the CS41 carbon after removal from the MCM-41 silica template (Figure 2c).

**Figure 3:**
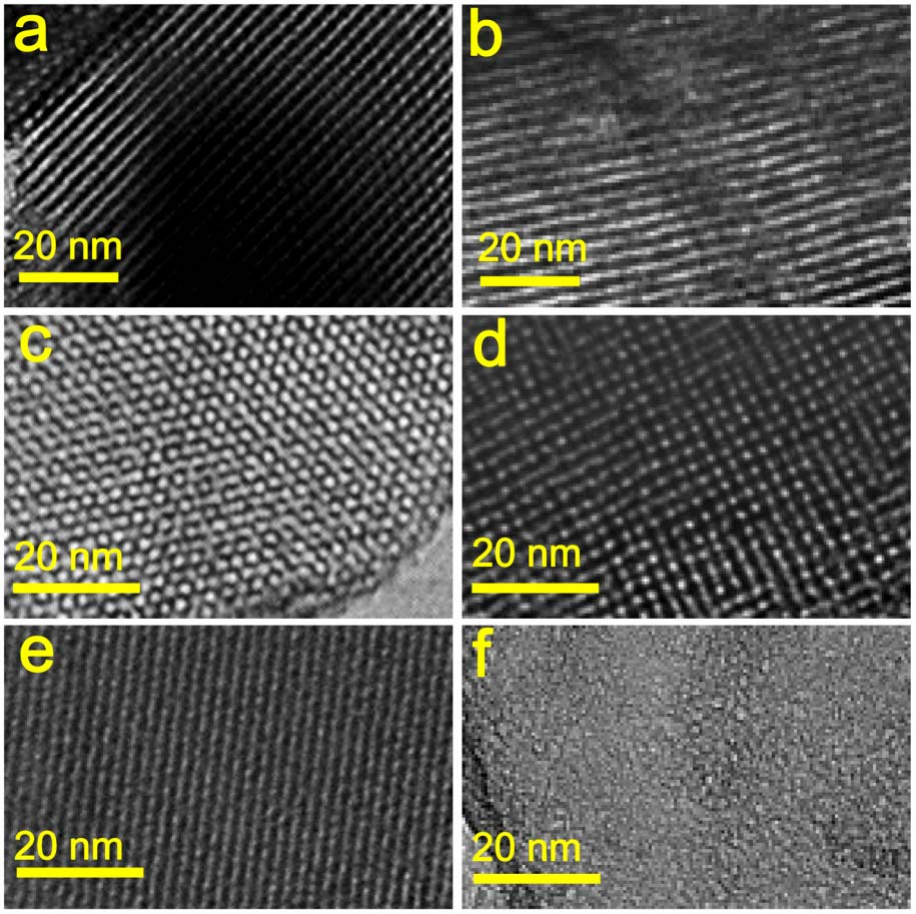
High-resolution TEM (HRTEM) images of the OMC/OMS intermedia with an inter-filled mesostructure. (a) CMK-3/SBA-15, (c) CMK-1/MCM-48, and (e) CS41/MCM-41, along with the corresponding naked carbon replicas (b) CMK-3, (d) CMK-1, and (f) CS41 after the silica templates were removed.

The transmission electron microscopy (TEM) images show that all OMS/OMC intermedia have well-defined, ordered, inter-filled mesostructures of carbon and silica (Figure 3a, c, and e). The TEM images of the OMC replicas after removal of the silica templates (Figure 3b, d, and f) are in agreement with the small-angle XRD patterns, directly confirming the mesostructure types of the OMCs: a 2D hexagonal mesostructure in CMK-3^24^, a 3D cubic mesostructure in CMK-1^26^, and a non-ordered structure in CS41^35^. The CMK-3 carbon with the 2D ordered carbon nanowire arrays is interconnected by randomly distributed nanorods. It can thus be considered a quasi-3D interconnected network^36^. However, there is no pore connectivity between hexagonal parallel mesopores in MCM-41 silica, resulting in a non-ordered structure of its carbon replica CS41, which aggregates many non-ordered carbon rods after removal from the MCM-41 template^35^. It is noteworthy that CS41 retains a 2D hexagonal order despite the non-connected nanowire arrays in the composite. This is due to the CS41/MCM-41 intermedium with the inter-filled mesostructure introduced into the silica matrix (Figure 3e). The nitrogen sorption measurements at −196°C (Supplementary Figure S1) indicate very high surface areas and large pore volumes for the OMCs (*e.g.*, 1329 m^2^/g and 1.35 cm^3^/g for CMK-3, 1282 m^2^/g and 0.96 cm^3^/g for CMK-1, and 1441 m^2^/g and 0.93 cm^3^/g for CS41) (Supplementary Table S1). As expected, the inter-filled OMC/OMS intermedia containing different OMCs have significant effects on the electric, dielectric, and EMI shielding properties of the ternary OMC/OMS/fused silica bulk composites.

The ternary OMC/OMS/fused silica composites from hot-pressing are easily compacted because of the viscous flow of silica during the sintering process^37^,^38^. The composites with CMK-3/SBA-15 and CMK-1/MCM-48 have a similar relative density of ~94% (Supplementary Table S2). The composite containing CS41/MCM-41 possesses the highest relative density of 95.8%. Thus, the influence of these materials on the EMI SE properties is as follows: CS41/MCM-41 > CMK-3/SBA-15 ≈ CMK-1/MCM-48. This order can be explained by the fact that the CS-41/MCM-41 intermedium filler provides less resistance to the flow of the molten silica matrix than does CMK-3/SBA-15 or CMK-1/MCM-48 during hot-pressing. The relative density of the composites is higher than the relative density of ~93% for CNT/fused silica composites containing the same carbon content^21^. This implies that the OMC/OMS particles are more homogeneously dispersed within the silica matrix than the large aspect ratio CNTs, which easily tangle and interact with each other. Moreover, the wide-angle XRD pattern demonstrates that the phase of the final sintered composite is amorphous (Supplementary Figure S2), indicating that no cristobalite formed during hot-pressing at 1300°C^38^. The crystallization of silica glass during high-temperature sintering degrades the properties of the composites.

**Figure 4:**
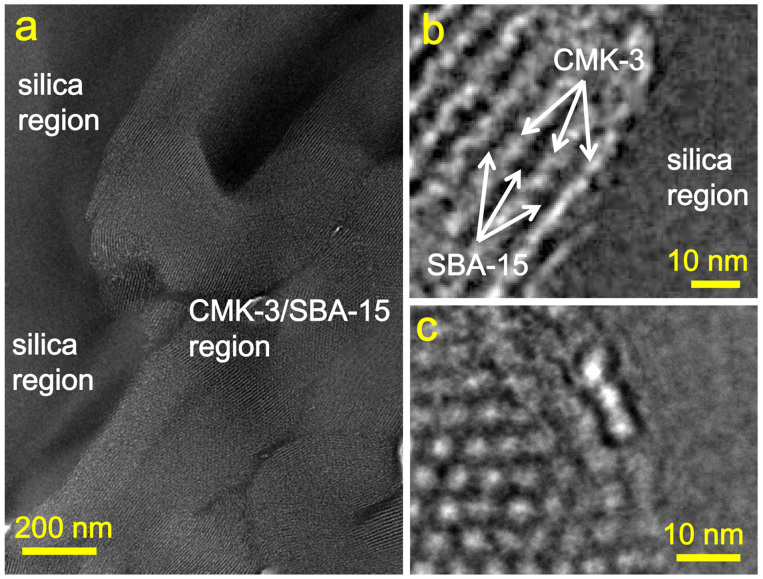
(a) TEM and (b-c) HRTEM images of the dense CMK-3/SBA-15/silica composite. The images were taken from (b) [100] and (c) [001] directions for CMK-3 carbon.

After densification of the inter-filled OMC/OMS intermedia into the silica matrix by hot-press sintering, the mesostructures of the OMCs in the sintered composites are retained. As shown in Figure 4, the microstructures of the OMC/OMS/fused silica composites can be characterized by TEM, taking the CMK-3/SBA-15-loaded composite as an example. The CMK-3/SBA-15 whole particles disperse randomly within the silica matrix. These particles are in close contact with the matrix. No obvious cavities are present in the bulk composite (Figure 4a). Therefore, the as-sintered OMC/OMS/fused silica ternary composites are very dense, in agreement with the density measurements. High-resolution TEM (HRTEM) images clearly indicate a 2D ordered mesostructure for the CMK-3/SBA-15 with an inter-filled structure. It is believed that the unique inter-filled mesostructure in the CMK-3/SBA-15 protects the CMK-3 carbon from being broken down. Similarly, it can be deduced that the CMK-1 and CS41 still possess ordered mesostructures in the dense composites, as observed in the CMK-1/MCM-48 and CS41/MCM-41 intermedia (Figure 3c, e).

**Figure 5:**
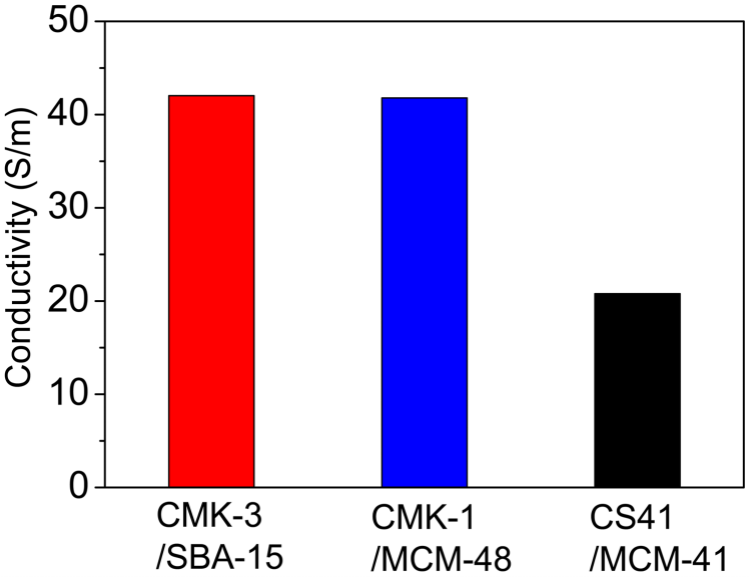
The electric conductivity of the ternary composites containing different inter-filled OMC/OMS particles.

The incorporation of inter-filled OMC/OMS particles into the silica matrix increases the electric conductivity of the composites. However, how the different OMC mesostructures affect the electric conductivity of the composites remains unknown. As shown in Figure 5, the CMK-3- and CMK-1-loaded composites have similar electric conductivity values of up to 42 S/m, substantially higher than the 20 S/m value for the CS41/MCM-41/fused silica composite. This is because the quasi-3D and 3D interconnected carbon nanowire networks in CMK-3 and CMK-1 may efficiently establish electric conduction pathways throughout the entire composite. Thus, the electric conductivity of the two composites increases immensely and is much higher than that of the composite filled with the 2D ordered, but non-connected carbon rods arrays (i.e., the CS41/MCM-41 intermedium). According to electromagnetic transmission line theory, the electrons moving *via* 3D electric conduction pathways exhaust much of their electromagnetic energy as heat, leading to attenuation of the electromagnetic energy. Therefore, it can be inferred that CMK-3- and CMK-1-loaded composites have higher EMI SE than composite containing the CS41 filler^17^.

**Figure 6:**
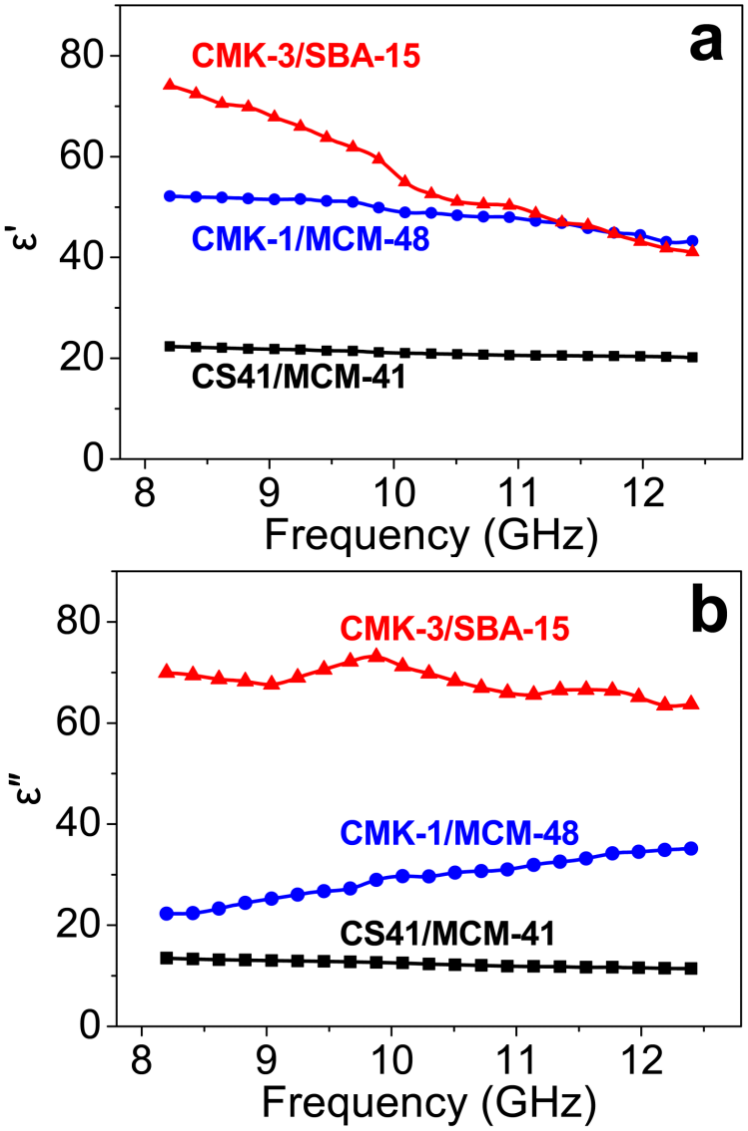
The complex permittivity spectra for the OMC/OMS/silica composites in the *X*-band frequency range. (a) Real permittivity (ε′). (b) Imaginary permittivity (ε″).

The complex permittivity of the composites, which is an important factor for EMI improvement, was also studied. Figure 6 shows the complex permittivity spectra in the *X*-band, including real (ε′) and imaginary (ε″) parts of the spectra, for the composites containing different OMCs. The results show that dielectric performance is highly sensitive to the OMC mesostructure. On average, both the ε′ and the ε″ values increase with the addition of OMCs in the following order: CS41-loaded < CMK-1-loaded < CMK-3-loaded. With loading of CS41, the ε′ value is *ca.* 21, 5.4 times higher than that of pure fused silica (dielectric constant: ~3.3 at 8–10 GHz)^39^. The ε′ rapidly increases up to 51 for the CMK-1-filled composite and reaches its highest value of 75 at a frequency of 10 GHz for the sample containing CMK-3. This rapid increase in the permittivity compared to that of pure fused silica is ascribed to the incorporation of the conductive carbon network^8^. The complex permittivity spectra of the CS41 composite shows a very slow, steady decrease in value with increasing frequency, whereas there are some fluctuations in both the real and the imaginary permittivity spectra for the composites containing CMK-3 and CMK-1. This implies a resonance behavior when the composite is highly conductive and the skin effect is significant^40^,^41^. The skin effect is the tendency for alternating current to flow near the surface of the conductor rather than over the entire cross-sectional area of the conductor. This phenomenon causes the resistance of the conductor to increase. Obviously, the resonance behavior is stronger in the 2D ordered carbon nanowire arrays of CMK-3 than in the 3D cubic mesostructure of CMK-1.

The ε″ value for complex permittivity is closely related to the dielectric loss^42^. For a higher ε″, there is a higher dielectric loss in the sample. As shown in Figure 6b, the ε″ values of all composites are much higher than 1. The highest ε″ value of *ca.* 72 at 9.8 GHz is obtained for the CMK-3-filled composite, while the CS41-composite exhibits the lowest ε″ value of 12. Although the composites with CMK-3 and CMK-1 have the same electric conductivity, the CMK-3-filled sample has a much higher ε″ value than that of the CMK-1-composite. The phenomenon can be attributed to the fact that the higher surface area and larger pore volume of CMK-3 provide a larger interfacial area. Thus, the more abundant potential localized defect sites in the heterogeneous interfaces between the carbon and silica in the CMK-3-filled composite act as permanent polarization centers, accounting for higher dielectric loss values^43^. The ε″ values for the CMK-3 and CS41-loaded composites decrease with increasing frequency (Figure 6b), as observed for the ε′ values in Figure 6a. However, the ε″ values of the CMK-1 composites show the reverse trend (*i.e.*, the ε″ gradually increases from 22 to 35 as the frequency changes from 8.2 to 12.4 GHz). This change is normally observed for composites with high electric conductivity^43^. In the present study, the unique 3D cubic mesostructure of CMK-1 can possibly lead to this feature, in contrast to the 2D (or quasi-3D) ordered carbon nanowire arrays of CMK-3. ε′ (electric polarization) and ε″ (electric loss) are correlated and the production of displacement current significantly lags behind the build-up of potential across the composite containing the 3D cubic carbon framework as the frequency increases^43^,^44^. Based on the fact that the mesostructures of the inter-filled OMC/OMS intermedia play crucial roles in changing the complex permittivity of the OMC-based composites, we reason that the mesostructures can further influence the EMI SE of these composites in the measured frequency range because the electromagnetic shielding properties are closely related to the permittivity according to transmission line theory.

**Figure 7:**
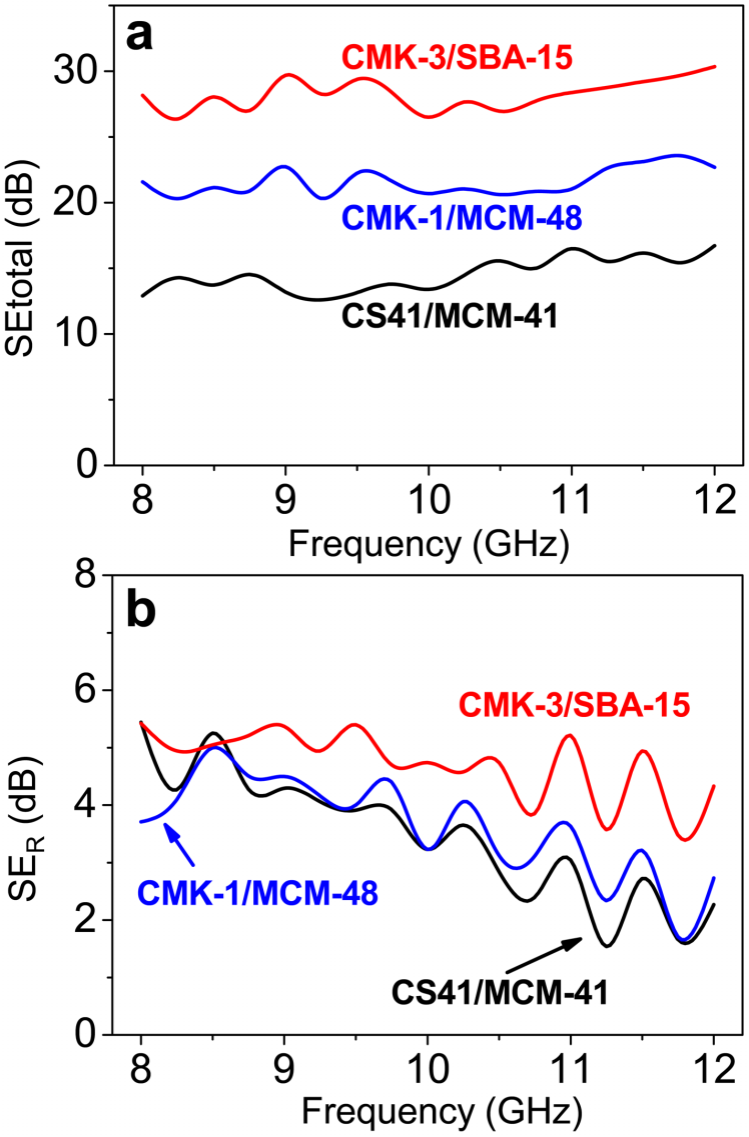
(a) The total EMI SE and (b) the reflection loss (SE_R_) of the 2.0 mm thick OMC/OMS/fused silica composites in the *X*-band frequency range.

The total EMI SE of a material can be defined by the following equation: SE_total_ = 10 log(*P_T_*/*P_I_*), where *P_T_* and *P_I_* are the power of the transmitted and incident EM waves, respectively^45^,^46^. The unit for EMI SE is the decibel (dB). The higher the SE value in dBs, the less energy passes through the material. Figure 7a shows the EMI SE of the OMC/OMS/fused silica composites as a function of frequency in the *X*-band. Pure fused silica is transparent to EM because of its insulating character. However, after the conducting OMCs are introduced, the average EMI SE of the composites is improved in a similar manner as the mesostructure changes observed for the complex permittivity: CMK-3-loaded (~28.2 dB) > CMK-1-loaded (~21.6 dB) > CS41-loaded (~14.5 dB) (Figure 7a). The EMI shielding is caused by the presence of OMCs in the composites. All measured EMI SE_total_ spectra are almost steady over the whole *X*-band region; however, they oscillate within the fluctuation range of *ca.* 4 dB. According to the theory of EM waves, this fluctuation can be ascribed to the matching relationship of the EMI SE and the frequency, as well as to the thickness of the composite, leading to the dependency of EMI SE on EM wave frequency. Such fluctuations have also been observed for pure fused silica^25^ and CNTs/fused silica composites^47^.

It has been reported that the EMI SE of a composite filled with carbon is positively correlated to the electric conductivity of the composite^48^,^49^. The propagation of EM waves in the composites leads to the directional motion of charge carriers in the OMC nanowire networks. Thus, the resulting oscillatory currents can consume the EM energy. The low EMI SE for the CS41-filled composite can be ascribed to its poor electric conductivity compared to that of CMK-3- and CMK-1-loaded composites^48^,^49^. Although the composites with CMK-3 and CMK-1 loadings have similar electric conductivity, the CMK-3-loaded composite has superior EMI SE. This is not in agreement with the EMI shielding theory, which shows that EMI SE increases with increasing electric conductivity^48^,^49^. This phenomenon can be explained by the fact that EMI SE is dependent not only on the electric conductivity but also on the dielectric properties of the composites. As mentioned above, the CMK-3-loaded composite has a higher dielectric loss due to more potential defect sites arising from the large surface area (1329 m^2^/g) of its interface with CMK-3 carbon and the SBA-15 silica template. The higher conductivity and dielectric loss promote EMI shielding for the CMK-3-filled composite. For commercial applications, the target EMI SE is *ca.* 20 dB (equal to only 1% transmission of the incident power)^50^. This means that the composites containing CMK-1 and CMK-3 can be used commercially as good EMI shielding materials, especially in harsh high-temperature or corrosive environments, because of their ceramic matrices.

The total EMI SE of a shielding material is the sum of the contributions of the absorbance of the EM energy (SE_A_), the reflection of the radiation (SE_R_), and the multi-reflection at various surfaces or internal interfaces (SE_M_): SE_total_ = SE_A_ + SE_R_ + SE_M_^45^. According to SchelKunoff 's theory, the loss ascribed to the multi-reflection (SE_M_) can be negligible if the shield is thicker than the skin depth (*δ*). The depth at which the field decreases to 1/*e* of the incident value is called the skin depth (*δ*), which can be written as the following equation^17^: 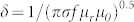
where *σ* is the electric conductivity of the conductor in S/m, *f* is the frequency of radiation in Hz, *μ*_0_ is the absolute permeability of the free space (4π × 10^−7^ H/m), and *μ_r_* is the relative permeability of the conductor. The term *μ_r_* can be taken as 1 because of the non-magnetic property of the OMC and silica. Therefore, it is apparent that the skin depth (*δ*) drops with increasing frequency (*f*) and conductivity (*σ*). At 8 GHz, the calculated *δ* value is 0.87 mm for both CMK-3- and CMK-1-filled composites and 1.26 mm for the CS-41-loaded composite. Both values are lower than the thickness (2 mm) of the measured OMC/OMS/fused silica composites, so SE_M_ can be neglected and the total EMI SE can be described by: SE_total_ ≈ SE_A_ + SE_R_. As shown in Figure 7b, all composites have low SE_R_ values. The average values are only ~4.7, 3.6, and 3.5 dB in the measured frequency region for the CMK-3-, CMK-1- and CS41-filled composites, respectively. Therefore, the contribution of SE_A_ to the SE_total_ is ~80% for the CMK-3- and CMK-1-loaded composites and ~75% for the composite with CS41. Absorption is the main contributor to the total EMI SE of the OMC-incorporated composites. In contrast, reflection is much higher than the absorption in CNT-dispersed EMI shielding materials^10^,^49^. Our experimental results imply that the ordered carbon mesostructures loaded within the matrix improve the absorption by multi-absorption from intra-re-reflecting and scattering of the incident EM wave in the confined space and at the interface of ordered OMC/OMS inter-filled mesostructures because of 3D interconnected conductive carbon nanowire networks. The SE_A_ value of the CMK-3-filled composite is over 20 dB in the *X*-band, implying its potential application as an EM absorbing material.

## Discussion

In conclusion, ternary dense bulk composites of OMC/OMS/fused silica have been prepared by in-situ nanocasting in combination with hot-pressing. The OMCs are characterized by high surface areas and large pore volumes. They can be formed with different mesostructure spatial configurations: 2D/quasi-3D hexagonal ordered carbon nanowire arrays interconnected by randomly distributed nanorods in the CMK-3-filled composite, 3D cubic mesostructure in the CMK-1-loaded composite, and 1D ordered isolated carbon nanowire arrays in the CS41-incorporated composite. Our results indicate that the texture and performance of these composites are strongly affected by the OMC mesostructure as follows for relative density (CS41-loaded (95.8%) > CMK-3-loaded (94.0%) ≈ CMK-1-loaded (94.4%)), electric conductivity (CMK-3-loaded (42 S/m) = CMK-1-loaded > CS41-loaded (20 S/m)), and complex permittivity (CMK-3-loaded > CMK-1-loaded > CS41-loaded). It is believed that the CS41/MCM-41 intermedium with 1D ordered non-connected carbon nanowires not only provides less resistance to the viscous flow of silica during densification, but also is less efficient for electron transport than the quasi-3D and 3D interconnected carbon frameworks in the CMK-3/SBA-15 and CMK-1/MCM-48 intermedia embedded in the silica matrix. Moreover, the total EMI SE of the OMC-based composites is correlated with the mesostructure spatial configurations as follows: CMK-3-loaded (28.2 dB) > CMK-1-loaded (21.6 dB) > CS41-loaded (14.5 dB). The low complex permittivity and EMI SE of the CS-41 composite are attributed to its poor electric conductivity. This shows that the CMK-3 possessing 2D hexagonally ordered nanowires in parallel arrays is more advantageous for EMI shielding compared to the 3D cubic mesostructure of CMK-1, possibly because of more potential defect sites from the larger surface area (1329 m^2^/g) of the interface for CMK-3 carbon and the SBA-15 silica template. Moreover, absorption mainly contributes to the total EMI SE in the OMCs-filled composites. The present results indicate that the CMK-3- and CMK-1-incorporated silica composites are highly effective EMI shielding materials in the *X*-band frequency range, especially under high temperatures or corrosive working conditions, offering advantages over various other polymer-based EMI shielding materials. The present research reveals a new method and concept for developing high-performance EMI shielding materials.

## Methods

### Materials

Sucrose, hydrofluoric acid (HCl), sulfuric acid (H_2_SO_4_), tetraethylorthosilicate (TEOS), and hexadecyltrimethylammonium bromide (C_16_TAB) were purchased from Sinopharm Chemical Reagent Co. Ltd. and Shanghai Lingfeng Chemical Reagent Co. Ltd., China. Ordered mesoporous silica SBA-15 powders were purchased from Fudan University. MCM-48 and MCM-41 were provided by Nanjing Xianfeng Nano Company. All chemicals were used as received without any further purification. Distilled water was used in all experiments.

### Filling the mesopores of ordered mesoporous silicas (OMS) (SBA-15, MCM-48, and MCM-41) with carbon *via* nanocasting

The OMC/OMS intermedia with an inter-filled mesostructure were prepared *via* a nanocasting strategy according to the literature^26^. Typically, OMS (1 g) was mixed with an aqueous solution (5 mL) containing calculated amounts of sucrose and H_2_SO_4_. The resulting mixture was heated in an oven at 100°C for 6 hours, followed by a further treatment at 160°C for another 6 hours to allow partial carbonization. To obtain fully polymerized and carbonized sucrose inside the mesopores of the OMS, an additional aqueous solution of sucrose and H_2_SO_4_ was added to the pretreated sample and the mixture was again subjected to the thermal treatment described above. Carbonization was completed by pyrolysis at 900°C under nitrogen flow, yielding black OMC/OMS intermedia.

### Preparing the OMC/OMS/fused silica bulk composites

To disperse the OMC/OMS intermedia particles into the silica matrix, a rapid sol-gel method was adopted using C_16_TAB as the surfactant^51^. The OMC/OMS composite powder was added to an aqueous solution containing C_16_TAB. Then, the solution was magnetically stirred for 2 h, followed by ultrasonication for another 1 h. Next, a measured amount of TEOS was slowly added to the solution while stirring before the dilute HCl solution was added drop-wise to adjust the pH value to 0 ~ 1. After the mixture was stirred for 3 h, which was sufficient to achieve complete hydrolyzation of TEOS, an ammonia solution was added to the mixture to initiate gelation. The gel was allowed to rest for one hour before being broken up by application of an exterior force. The gel was then washed with large quantities of distilled water, as well as with ethanol, to remove any impurities. After being dried at 110°C overnight, the OMC/OMS/silica composite powder was further calcinated at 300°C for 1 h to remove any residual surfactant and improve the condensation of the silanol groups by dehydration. Finally, a hot-pressing technique was used to sinter the OMC/OMS/SiO_2_ composites. The composite powder was loaded into a graphite die with a cylinder chamber (diameter: 25 mm). The hot-pressing process was conducted under an argon atmosphere in a multi-purpose furnace. Samples were rapidly heated to 1300°C at a rate of 50°C/min and a pressure of 16 MPa. The samples were then held under 35 MPa pressure for 30 min. When the samples started to cool, the pressure was released. The OMC/OMS/fused silica bulk composites were then removed from the mold.

### Characterization techniques

X-ray powder diffraction (XRD) patterns were collected on a Rigaku D/MAX-c β instrument using Cu Kα_1_ (λ = 0.15406 nm) radiation at 40 kV and 60 mA. Nitrogen adsorption-desorption isotherms were measured on a Micromeritics ASAP2010 surface area and pore size analyzer at liquid nitrogen temperature (−196°C). Prior to measurements, the samples were dehydrated at 100°C and then outgassed at 200°C in a vacuum for 4 h. The Brunauer-Emmett-Teller (BET) method was used to calculate the specific surface areas (S_BET_). The pore volume (V_BJH_) and the mean pore size (D_BJH_) were derived from the adsorption branches of the isotherms using the Barrett-Joyner-Halanda (BJH) method. The density of the composites was measured by Archimedes' method. The density was tested for six samples from each concentration and reported as the average. TEM images were acquired on a JEOL 2010 CX electron microscope operating at 200 kV. To observe the carbon nanostructure of the composites, a small piece of the OMC/OMS/fused silica was abraded to a thickness of several micrometers, followed by post-treatment with an ion beam thinner. A Hall Effect Measurement System (Lake Shore 7704A) was used to measure the electric conductivity of the composites at room temperature. The electric conductivity was tested for six samples from each concentration and reported as the average. The relative permittivity was measured using a vector network analyzer (HP 8722ES) in the *X*-band frequency range. In preparation for the electromagnetic interference (EMI) shielding measurements, the composites were cut to 22.9 × 10.2 × 2.0 mm^3^ in size. The EMI shielding effectiveness of the samples was measured at room temperature using an Agilent N5242A microwave network analyzer following industry standard procedures. The microwave shielding properties were deduced by calculations based on the transmission line theory related to the experimentally measured permittivity.

## Author Contributions

J.W. wrote and edited the manuscript; H.Z. and J.Z. conducted the experiments and analyzed the data; and Q.L. designed the experiments and commented the manuscript.

## Supplementary Material

Supplementary Information supporting information.pdf

## Figures and Tables

**Figure 1:**
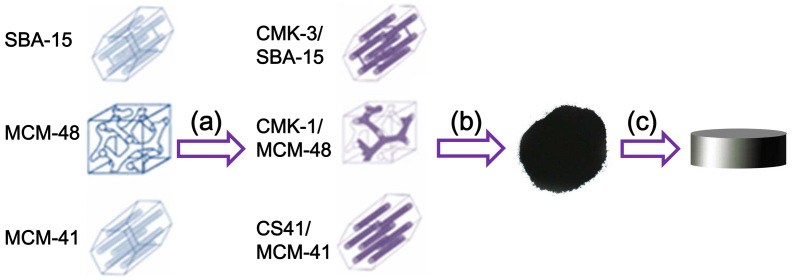
Schematic diagram showing the synthesis of ternary composites of OMC/OMS/fused silica with 10 wt% carbon loading. (a) In-situ filling of OMS with carbon and formation of the OMC/OMS intermedium with an inter-filled mesostructure. (b) Combination of the as-synthesized OMC/OMS rod-like particles and a mixture of tetraethyl orthosilicate (TEOS) and hydrochloric acid (HCl), followed by gelling, drying, and calcination to prepare the ternary OMC/OMS/silica composite powders containing OMCs with different mesostructures. (c) Hot-pressing was used to prepare the OMC/OMS/fused dense silica bulk composites from the OMC/OMS/fused silica composite powders.
