# Supplementary material for: Influence of spatial configurations on electromagnetic interference shielding of ordered mesoporous carbon/ordered mesoporous silica/silica composites
Source: Sci Rep. 2013 Nov 19;3:3252. doi: 10.1038/srep03252 (PMC3832851; doi:10.1038/srep03252)
Supplement: Supplementary Information — supporting information.pdf [file srep03252-s1.pdf]

Influence of spatial configurations on electromagnetic interference shielding of ordered mesoporous carbon/ordered mesoporous silica/silica composites

Jiacheng Wang,*[a] Hu Zhou,[a,b] Jiandong Zhuang[a] and Qian Liu*[a]

[a] State Key Laboratory of High Performance Ceramics and Superfine Microstructure, Shanghai Institute of Ceramics, Chinese Academy of Science, Shanghai 200050, P. R. China. Tel: 0086-21-52412612. Fax: 0086-21-52413122; Email address: jiacheng.wang@mail.sic.ac.cn; qianliu@sunm.shcnc.ac.cn

[b] University of Chinese Academy of Sciences, Beijing 100049, P. R. China.

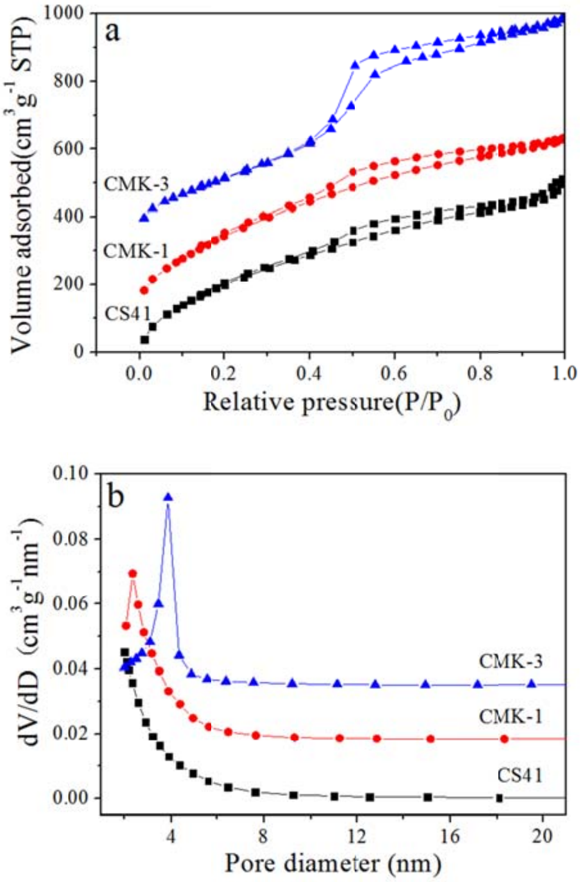

Figure S1. a) Nitrogen adsorption-desorption isotherms and b) pore size distributions of the resulting various OMC replicas (CMK-3, CMK-1, and CS41).

Table S1. Textural properties of various OMC replicas: CMK-3, CMK-1, and CS41.

	S_{BET} (m^2/g)	V_{BJH} (cm^3/g)	D_{pore} (nm)
CMK-3	1329	1.35	4.2
CMK-1	1284	0.96	2.9
CS41	1441	0.93	< 2

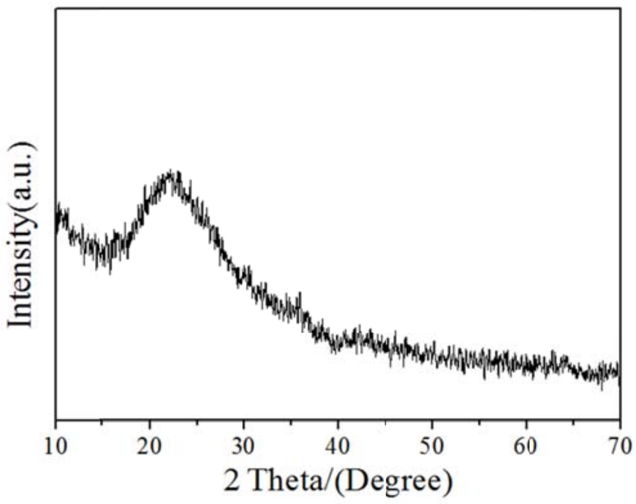

Figure S2. Wide-angle XRD pattern of the CMK-3/SBA-15/fused silica composite bulk.

Table S2. Relative density of OMC/OMS/fused silica composite bulks.

Sample	Relative density
CMK-3/SBA-15/SiO_2	94.0 %
CMK-1/MCM-48/SiO_2	94.4 %
CS41/MCM-41/SiO_2	95.8 %
